# Analysis of data from the PALOMA-3 trial confirms the efficacy of palbociclib and offers alternatives for novel assessment of clinical trials

**DOI:** 10.1007/s10549-023-07131-7

**Published:** 2023-11-13

**Authors:** Celine Yeh, Mengxi Zhou, Neil Bapodra, Dawn Hershman, Edward Espinal, Marina Moran, Maria Rivero, Antonio Tito Fojo, Susan E. Bates

**Affiliations:** 1 Department of Medicine, Division of Hematology Oncology, Columbia University College of Physicians and Surgeons, New York, NY USA; 2 Department of Radiology, Columbia University College of Physicians and Surgeons, New York, NY USA; 3 James J. Peters VAMC, Bronx, NY USA; 4 Pfizer España, Avenida de Europa, 20 – B-Parque Empresarial. La Moraleja, 28108 Alcobendas (Madrid), Spain

**Keywords:** PALOMA, CDK4/6, CDK4/6 inhibitors, Abemaciclib, Ribociclib, Overall survival

## Abstract

**Purpose:**

There remains a need for novel therapies for patients with metastatic breast cancer (MBC). We explore the use of a novel biomarker of survival that could potentially expedite the testing of novel therapies.

**Methods:**

We applied a tumor regression-growth model to radiographic measurement data from 393 women with MBC enrolled in PALOMA-3 examining efficacy of palbociclib in disease that had progressed on previous endocrine therapy. 261 and 132 women were randomized to fulvestrant plus palbociclib or placebo, respectively. We estimated rates of regression (***d***) and growth (***g***) of the sensitive and resistant fractions of tumors, respectively. We compared the median ***g*** of both arms. We examined the relationship between ***g*** and progression-free and overall survival (OS).

**Results:**

As in other tumors, ***g*** is a biomarker of OS. In PALOMA-3, we found significant differences in ***g*** among patients with tumors sensitive to endocrine therapy but not amongst resistant tumors, emulating clinical trial results. Subgroup analysis found favorable ***g*** values in visceral metastases treated with palbociclib. Palbociclib efficacy demonstrated by slower ***g*** values was evident early in the trial, twelve weeks after the first 28 patients had been enrolled.

**Conclusion:**

Values of ***g***, estimated using data collected while a patient is enrolled in a clinical trial is an excellent biomarker of OS. Our results correlate with the survival outcomes of PALOMA-3 and argue strongly for using *g* as a clinical trial endpoint to help inform go/no-go decisions, improve trial efficiency, and deliver novel therapies to patients sooner.

**Supplementary Information:**

The online version contains supplementary material available at 10.1007/s10549-023-07131-7.

## Introduction

United States Breast Cancer Statistics estimate one of every eight women will develop breast cancer during her lifetime. In 2022, it is estimated 287,850 women will have a new diagnosis of invasive breast cancer with an additional 51,400 new diagnoses of non-invasive (in situ) breast cancer [1]. While 5-year relative survival rates of women with any stage of breast cancer is 90% and local disease is 99%, the 5-year rates with a regional presentation or distant disease are only 86% and 28% respectively [2]. Worldwide, female breast cancer surpassed lung cancer as the most diagnosed cancer in 2020, with an estimated 2.3 million new cases, and 690,000 deaths—11.7% and 6.9% of all cancers and cancer deaths, respectively. Importantly, the overall incidence of breast cancer deaths was considerably higher in transitioning versus transitioned countries with incidences of 15.0 vs 12.8 per 100,000, respectively [3]. While advancements have led to higher survival rates, there remains a need for novel therapies. Beginning with palbociclib, cyclin-dependent kinase 4 and 6 (CDK4/6) inhibitors [4, 5] have emerged as an option with their clinical activity and especially their toxicities anchored on their cell cycle effects.

Flavopiridol, a first generation non-selective CDK inhibitor proved effective but was eventually deemed too toxic [6–8]. Subsequent to flavopiridol, three selective CDK4/6 inhibitors—palbociclib, ribociclib, and abemaciclib—have secured regulatory approvals in breast cancer [9–18]. Palbociclib, a well-tolerated and effective CDK4/6 inhibitor was initially granted accelerated approval in February 2015, in combination with letrozole for the treatment of estrogen receptor (ER)-positive, HER2-negative advanced breast cancer [19]. The accelerated approval as initial endocrine-based therapy in postmenopausal women leveraged the results of the PALOMA-1 trial [11]. In February 2016, the FDA granted palbociclib regular approval in combination with fulvestrant for the treatment of HR-positive, HER2-negative advanced or metastatic breast cancer (MBC) in women with disease progression following endocrine therapy, this time relying on the results of the PALOMA-3 clinical trial [9, 17, 18, 20, 21]. Similar approvals have been granted by the EMA and other regulatory agencies [19].

In oncology clinical trials, OS has been established as the most reliable and preferred endpoint, although it needs a long follow-up period in large trials, and subsequent cancer treatments can affect the results [22]. In the luminal population (HR + /HER2−), the OS endpoint is hard to achieve as long survival times are expected in this disease, and progression-free survival (PFS) has historically been chosen as an appropriate endpoint in randomized trials for hormonal drugs [23]. Validation of novel surrogate endpoints of OS in luminal breast cancer trials could facilitate the approval of new cancer drugs and accelerate the access of novel drugs to patients.

In contrast to the two first-line studies, PALOMA-3 enrolled patients who had been previously treated with endocrine therapy (on up to fourth line of endocrine therapy for MBC), and stratified patients based on sensitivity to prior endocrine therapy. Using data obtained exclusively while patients were enrolled in the trial, we estimated the rates of tumor growth (***g***) during therapy. Relying on extensive experience with our method of analysis and repeated demonstration that ***g*** is a biomarker of OS [24–29], we have ratified the PALOMA-3 results and now add breast cancer to the list of tumors where ***g*** can be explored and leveraged as a valid biomarker of OS.

## Methods

### Mathematical model

The model assumes that changes in tumor quantity during treatment result from two processes—exponential ***d***ecay of the treatment-sensitive fraction of tumor at rate ***d***, and exponential ***g***rowth or re-growth of the resistant fraction at rate ***g*** (Supplemental Fig. 1). The data of most tumors can be fitted to one of four equations:

1 $$f\left( t \right)\, = \,{\text{exp}}\left( { - \,d \bullet t} \right)\, + \,{\text{exp}}\left( {g \bullet t} \right)\, - \,{1}$$

2 $$f\left( t \right)\, = \,{\text{exp}}\left( {g \bullet t} \right)$$

3 $$f\left( t \right)\, = \,{\text{exp}}\left( { - \,d \bullet t} \right)$$

4 $$f\left( {\text{t}} \right)\, = \theta \,{\text{exp}}\left( { - \,d \bullet t} \right)\, + \,({1}\, - \,\theta )\,{\text{exp}}\left( {g \bullet t} \right)$$

*f(t)* represents tumor burden at time *t* (in days), relative to a quantity of 1 for radiographic measurements at *t* = 0. ***g*** (in days^−1^) is the rate of ***g***rowth and is related to tumor doubling time (*T*_*d*_) by the formula *T*_*d*_ = 0.693/***g***, where 0.693 is the natural logarithm of 2. ***d*** (in days^−1^) is the rate of ***d***ecay. ${\varvec{\theta}}$ is the treatment-sensitive fraction of tumor and (1– ${\varvec{\theta}}$) the fraction absolutely or relatively resistant to treatment.

The basic model ***gd*** is described by Eq. (1). In cases where the data demonstrate only an increase in tumor burden from the beginning of therapy (i.e., only ***g*** differs significantly from 0 with *p* < 0.1), ***d*** is eliminated, and tumor ***g***rowth rate is estimated using the ***gx*** equation [Eq. (2)]. Similarly, in cases where the data demonstrate only a reduction in tumor burden from the beginning of therapy (i.e., only ***d*** differs significantly from 0 with *p* < 0.1), ***g*** is eliminated, and tumor ***d***ecay rate is estimated using the ***dx*** equation [Eq. (3)]. ${\varvec{\theta}}$ represents the proportion of tumor that is sensitive to therapy. In cases where the data allow the estimation of three parameters, ${\varvec{\theta}}$ can be estimated using the ***gd*** ${\varvec{\theta}}$ equation [Eq. (4)]. Incorporation of time (*t*) in the equations renders the analysis indifferent to time (i.e., intervals of assessment used by the study).

### Data analysis

The *tumgr* package [30] in R sequentially applies Eqs. 1–4 and selects the equation that best fits the tumor’s data. In this study, tumor ***g***rowth and ***d***ecay rates were derived by entering serial radiographic tumor measurements into the *tumgr* package. Values for ***g***, ***d***, or ${\varvec{\theta}}$ were estimated only if the fit of the data had a *p*-value of < 0.1 (Supplemental Fig. 2). In cases where all parameters were significant predictors of tumor quantity in more than one model, the model that best minimized the Akaike Information Criterion (AIC) was selected, with AIC = [(− 2 ⋅ log likelihood of model) + (2 ⋅ number of parameters in model)].

Comparisons of the distributions of ***g*** and ***d*** based on treatment arm or other variables were made using non-parametric *t*-tests. The Kaplan–Meier (KM) method was used to plot PFS and OS.

Analyses were performed in R version 3.6.1 using the *tumgr* package. Survival analyses were performed using the *survival* package [31].

### Data sources

Trial data for PALOMA-3 were provided by Pfizer, Inc. The data provided included uncensored data from 393 patients enrolled on trial who had measurable tumor. Supplemental Table 1 summarizes the characteristics of the trial.

**Table 1:** Summary of the data analysis for the PALOMA-3 trial

	All data	Resistant		Sensitive	
		Fulvestrant + Placebo (*n* = 31)	Fulvestrant + Palbociclib (*n* = 56)	Fulvestrant + Placebo (*n* = 101)	Fulvestrant + Palbociclib (*n* = 205)
Patients with sufficient data*	335 (85.2%)	24 (77%)	47 (84%)	83 (83%)	181 (88%)
dx	63 (18.8%)	1 (4%)	7 (15%)	12 (14%)	43 (24%)
gdphi [gd $\theta$]	29 (8.6%)	3 (13%)	9 (19%)	5 (6%)	12 (7%)
gd	146 (43.6%)	10 (42%)	20 (43%)	31 (37%)	85 (47%)
gx	68 (20.4%)	6 (25%)	8 (17%)	27 (33%)	27 (15%)
Total fit of those with sufficient data	306 (91.4%)	20 (83%)	44 (94%)	75 (90%)	167 (92%)
Not fit to any of the four models	29 (8.6%)	4 (17%)	3 (6%)	8 (10%)	14 (8%)

^*^Datasets were excluded for insufficient data defined as having no measurement, only one measurement or only two measurements [baseline + one more] with a difference of less than 20%

## Results

**Fig. 1:**
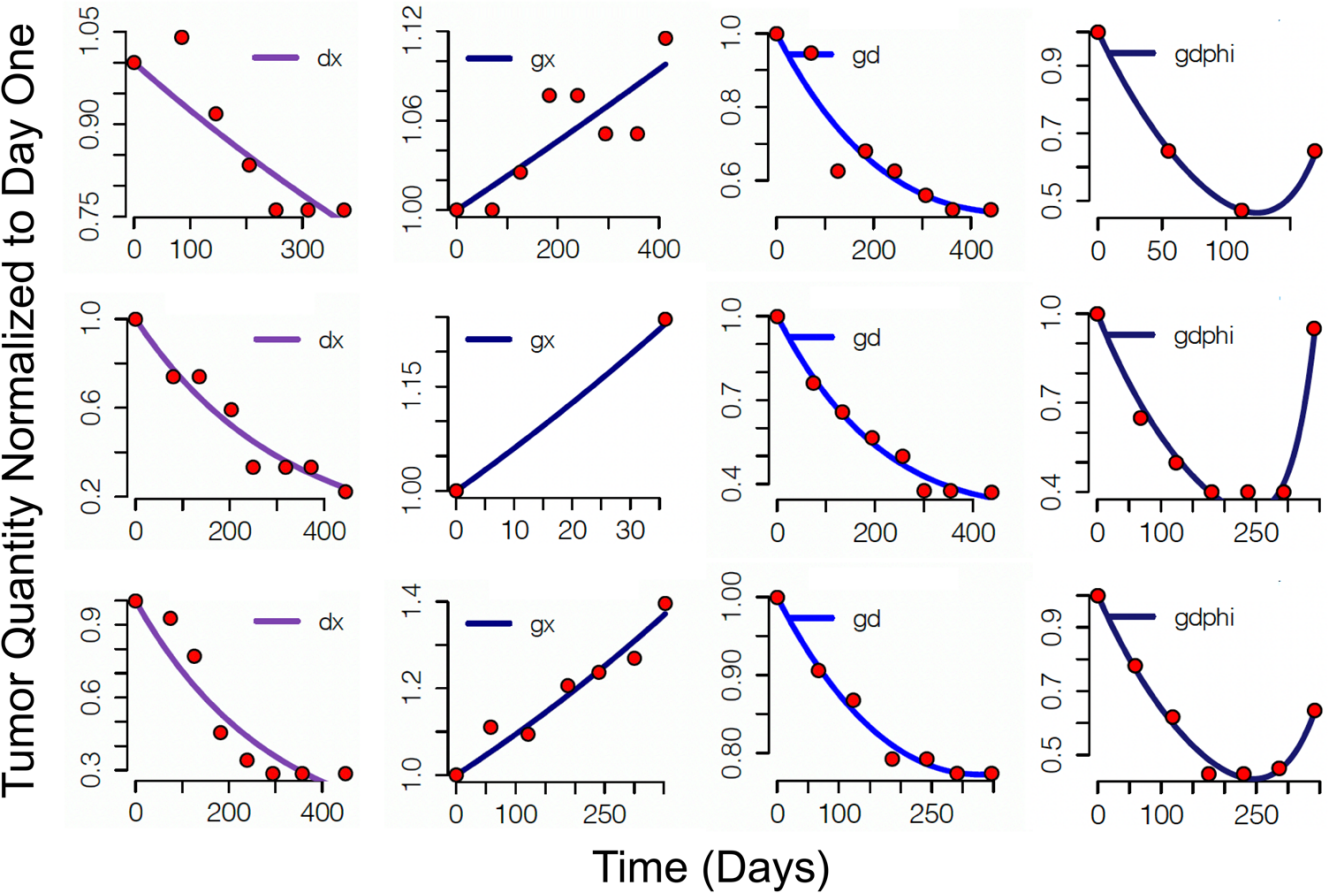
Curve fits to the data that were fit best by the *dx, gd, gd,*${\varvec{\theta}}$and *gx,* equations. The *y*-axis represents the quantity of tumor at a given time during treatment relative to the quantity of tumor at the initiation of treatment. The curve fits are derived from the fixed rate constants (***g***, ***d***, ${\varvec{\theta}}$), which themselves are estimated by an iterative process that evaluates the fit of the tumor’s data to Eqs. 1–4, and then selects the equation of best fit. Note the blue lines are “drawn” by calculating the tumor quantity across the range of times in the X-axis using the equation selected as best able to describe the data, and that equation’s estimated values of ***g***, ***d***, and ${\varvec{\theta}}$

We analyzed the data of 393 patients enrolled in PALOMA-3 (Supplemental Table 1). Best curve fits of individual cases are included in Fig. 1. Each illustrated case was fit best by one of the four models described earlier: ***gx, gd, gdf,*** or ***dx.***

**Fig. 2:**
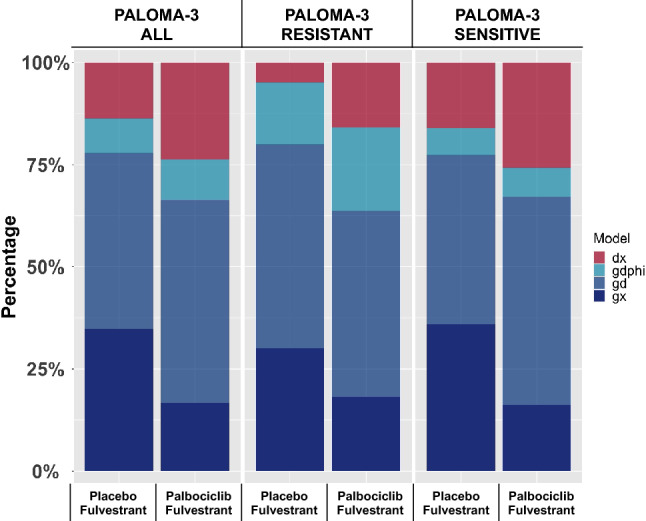
Distribution of equations best fit to radiographic tumor measurement data, by study treatment arm

Table 1 summarizes the data analysis for the trial. Radiographic tumor measurements were available for 261 from the experimental arm (fulvestrant in combination with palbociclib), and 132 from control arms (fulvestrant with placebo). Of the 393 patients, 58 (14.8%) had insufficient data—either no measurements, only baseline data, or only baseline plus one additional measurement with a difference less than 20%—and were not included in our analysis, leaving 335 (85.2%) patients with data sufficient for analysis. Values for either ***g*** or ***d*** could be determined for 306 (91.4%) of 335 patients with evaluable data. To be included in the analysis, the fit of the data had to conform to a *p*-value of $\le$ 0.1 and as shown in Supplemental Fig. 2, median *p*-values were orders of magnitude lower. Figure 2 displays distribution of equations that best fit data in each study arm. Note the data of more tumors were fit best by the ***gx*** model in the placebo than in the experimental arm—the ***gx*** fit describing the kinetics of tumors in which regression could not be detected. Lower percentages in the experimental arms would be expected with effective therapies.

**Fig. 3:**
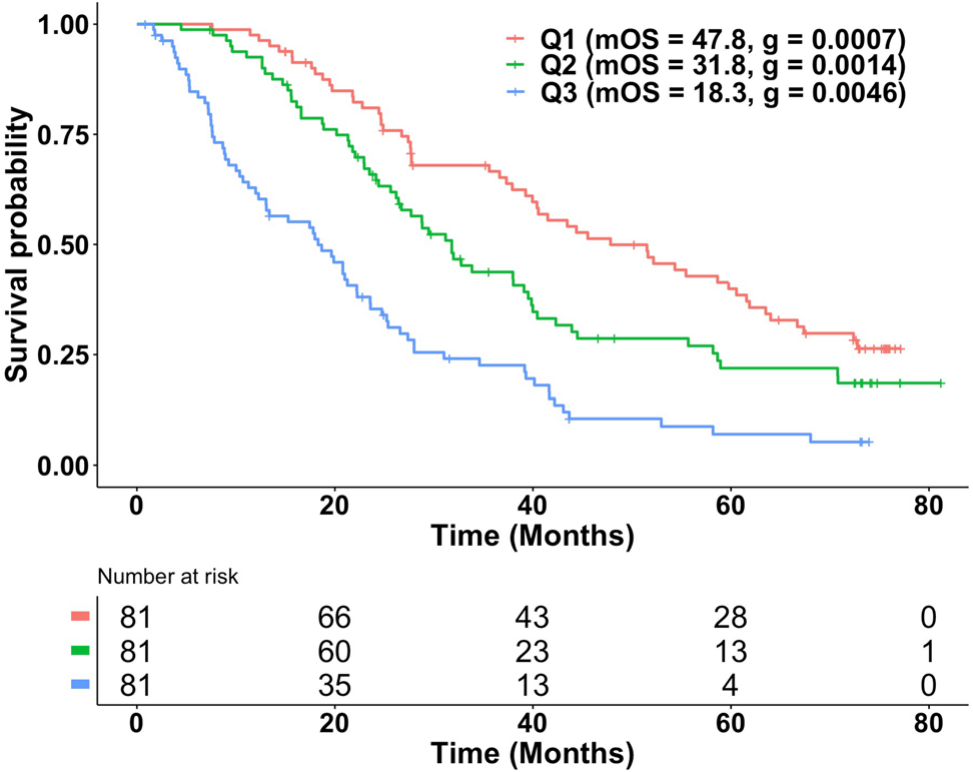
Kaplan–Meier plots of OS by tertile of the ***g*** predictor are displayed for estimates of ***g*** obtained using radiographic tumor measurement data from PALOMA-3. The graphic displays results that support ***g*** as a biomarker of overall survival

Previous studies have supported the use of ***g*** as a biomarker of OS [24–29]. Consequently, we assessed the extent to which ***g***, *estimated using only tumor measurement data obtained while enrolled on study*, could serve as a biomarker of OS occurring often long after clinical trial enrollment. OS values were available for 243 patients in whom a value of ***g*** had been determined—data best described by the ***gx***, ***gd*** or ***gd*** ${\varvec{\theta}}$ equations. Figure 3 shows Kaplan–Meier (KM) plots of OS by tertiles of ***g***. The first tertile (Q_1_) represented by the KM plot to the far right consists of patients whose tumors had the lowest ***g*** values—a median ***g*** of 0.0007. The median OS for this subset of patients was 47.8 months. The third tertile (Q_3_) represented by the KM plot to the far left consists of patients whose tumors had the highest ***g*** values—a median ***g*** of 0.0046. The median OS for this subset of patients was 18.3 months. The intermediate tertile lies in between. Thus, here, as in other cancers, ***g*** is a biomarker for OS. Finally, in an additional 63 women, the data were best fit by the ***dx*** equation that describes kinetics of tumors without detectable growth and no ***g*** value determined. The OS of these patients might be expected to be favorable, and this is shown in Supplemental Fig. 3 where the KM plot of the ***dx*** population overlaps with that of the best ***g*** tertile.

**Fig. 4:**
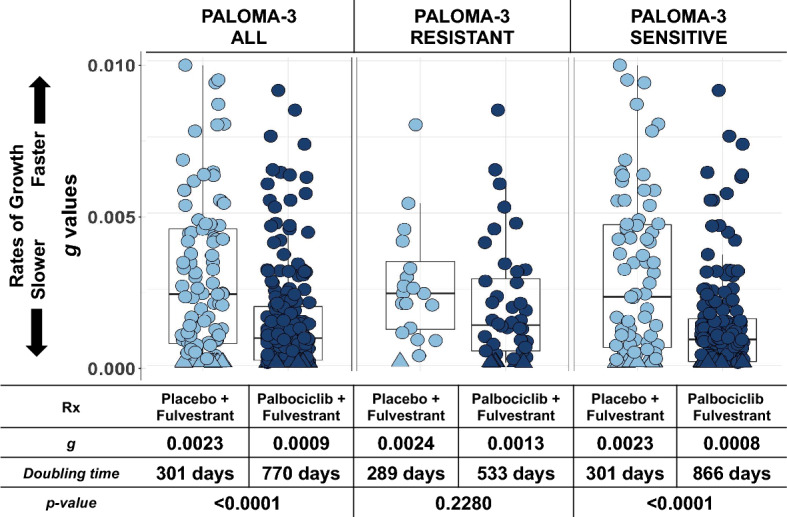
Graphical depiction of the distribution of ***g***, derived from radiographic tumor measurement data, by study treatment arm. Error bars show IQR × 1.5. Note how a statistical difference was achieved for the entire PALOMA-3 data [ALL] and the sensitive cohort, results consistent with the mature OS data. Note also very low ***g*** values in some patents in the resistant cohort, likely reflecting the presence of palbociclib activity in the residual sensitive cancer cells. This includes patients in whom a*** g*** could not be determined because their data fit the ***dx*** equation best. As shown in Supplemental Fig. 3, patients whose data were best fit by the ***dx*** equation had an overall survival comparable to the tertile with the lowest ***g*** values and in this analysis, a fixed low value of ***g*** (10^–4^) has been imputed for these tumors. The imputation lowers the median value of both cohorts to some extent but their inclusion with imputed values does not alter the results that are largely driven by those in whom a ***g*** value could be estimated

Given our results in this and other studies [24–29] establishing ***g*** as a biomarker of OS, we compared values of ***g*** for the different arms and subgroups in PALOMA-3. Figure 4 displays the distribution of ***g*** by study arm, imputing a fixed low value of ***g*** (10^–4^) for the tumors fit best by ***dx***. Consistent with published observations, we found significant differences in ***g*** between tumors treated with palbociclib plus fulvestrant when compared to those treated with fulvestrant plus placebo both in the entire data set and in the subset of tumors deemed sensitive to previous endocrine therapy (***g*** values of 0.0009 vs. 0.0023, and 0.0008 vs. 0.0023 in the experimental and control arms, respectively; *p* < 0.0001). Thus, estimates of ***g*** across PALOMA-3 tracks with the reports of OS.

**Fig. 5:**
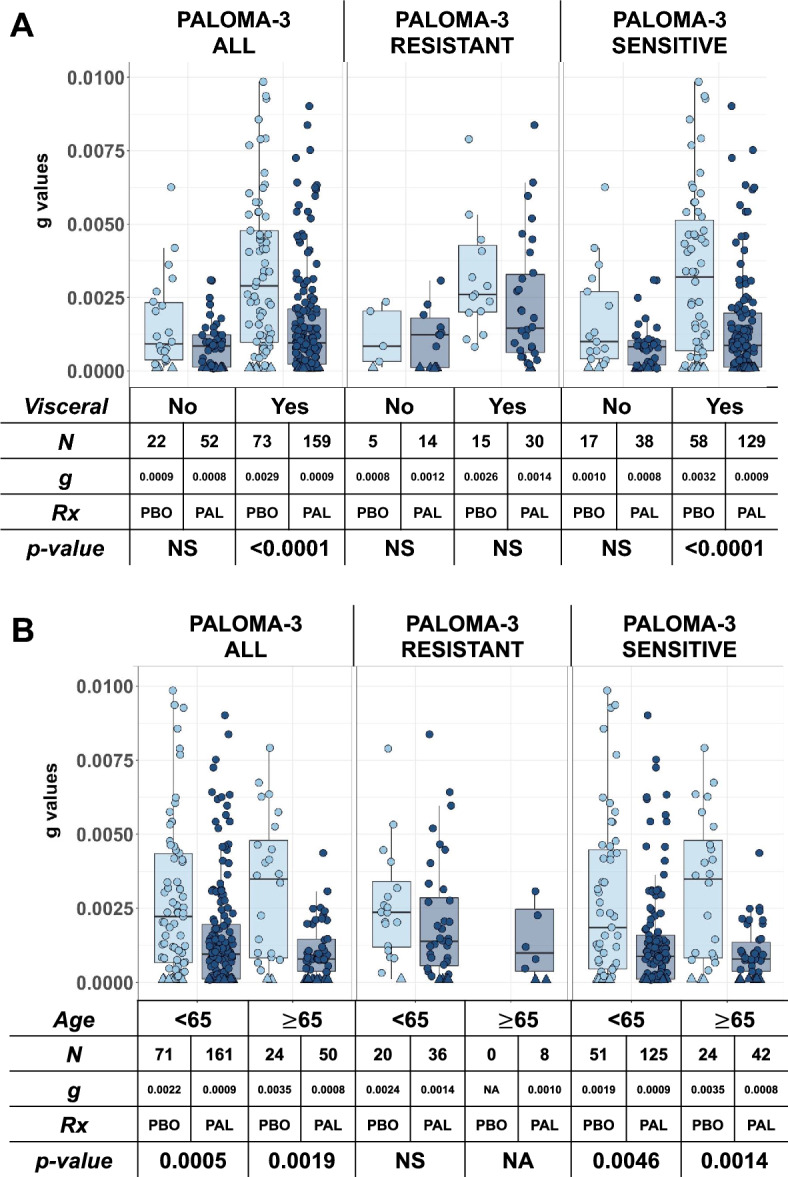
Graphical depiction of the distribution of ***g***, derived from radiographic tumor measurement data, by study treatment arm looking at sites of visceral disease (**A**) and according to age < 65 years or ≥ 65 years (**B**). Error bars show IQR × 1.5. Note again how a statistical difference was achieved for the entire PALOMA-3 data [ALL] and the sensitive cohort, with the addition of palbociclib but only at sites of visceral involvement and across age groups

Finally, we performed subgroup analyses based on the presence of visceral metastatic disease and age. Figure 5A displays the distribution of ***g*** values across the trial, further stratified into the presence/absence of visceral metastases. In PALOMA-3, we found a significant difference in ***g*** between experimental and control arms when examining the entire data set and in endocrine-sensitive tumors with evidence of visceral metastatic disease. Figure 5B displays the distribution of ***g*** across the trial, further stratified into age < 65 versus ≥ 65 years. In PALOMA-3, there was a significant difference in median ***g*** values between the experimental and control arms when examining the entire data set and the endocrine-sensitive tumors according to age [21]. Additional analyses examining menopausal status, ECOG performance status, disease-free interval and prior lines of therapy are shown in Supplemental Figs. 4–7.

## Discussion

Using a novel method of data analysis that models the simultaneous growth and regression of tumors using radiographic measurements, we analyzed the data of 393 patients with HR-positive, HER2-negative advanced breast cancer enrolled in PALOMA-3 [9–12, 17, 18]. We find as we have in other tumors [24–29] that the estimated rate of tumor growth, designated ***g***, is a biomarker of OS. Given this, we would expect ***g*** to differ significantly between the arms of any randomized trial that demonstrated a significant OS difference. In PALOMA-3, we found significant differences in ***g*** comparing all study participants and in patients whose tumors were sensitive to previous endocrine therapy, but not amongst those deemed to have intrinsic endocrine resistance, emulating the clinical trial results. Within the endocrine-sensitive subgroup of PALOMA-3, further subgroup analysis showed that a difference in ***g*** was observed in sites of visceral metastases (Fig. 5A), providing evidence of palbociclib efficacy against disease sites generally considered more difficult to treat [32]. Additionally, within the endocrine resistant subgroup, activity was also found in a subset of patients, most likely reflecting the presence of palbociclib activity in the residual sensitive cancer cells.

Given ***g*** is estimated using only data captured while a patient is enrolled on study, its emergence as a biomarker of OS, an event that occurs long after treatment is discontinued, argues strongly for both drug sensitivity *and* tumor biology as factors that contribute to the estimated rates of tumor growth.

Because all four equations include time as a variable, the interval of assessment is rendered irrelevant and hence, comparisons across trials can be made. In addition to palbociclib, other phase III trials have evaluated the efficacy of CDK4/6 inhibitors in combination with fulvestrant in patients with HR-positive, HER 2-negative advanced breast cancer whose disease had progressed on endocrine therapy. MONARCH-2 [16, 33] evaluated abemaciclib, while MONALEESA-3 [14, 15] assessed ribociclib. Using the growth rate metrics, cross trial comparisons could be performed that would help inform the utility of the three CDK 4/6 inhibitors, comparisons currently not possible. For example, while fulvestrant plus placebo was the control cohort therapy in PALOMA-3 [9], MONARCH-2 [16], and MONALEESA-3 [15], it is clear the patient populations were far from identical with differences in the inherent biology of the patient’s tumors and not the drugs likely driving the disparate trial outcomes. Median PFS values of control arms of 4.6, 9.3 and 12.8 months for palbociclib, abemaciclib, and ribociclib, respectively, provide clear evidence that the tumors of women who enrolled in PALOMA-3 had a more aggressive disease biology. This disparity readily explains the OS differences, an observation supported by the comparable hazard ratios for both PFS and OS. While these clear differences amongst the trials preclude definitive conclusions, the comparable hazard ratios for OS demonstrate that in a more difficult patient population, palbociclib performed as well as abemaciclib and ribociclib—an attribute we confirm by demonstrating comparable efficacy in those with/without visceral involvement—a known adverse site of involvement (Fig. 5A) [32]. Additionally, Supplemental Fig. 8 provides a look at the distribution of ***g*** values and shows the impact of palbociclib effectively lowering the growth rates (i.e., the distribution of ***g*** values in the histogram is narrowed by slowing those with faster rates—a recurrent observation in our analyses of data where an effective regimens is used). The inferred doubling time, equal to 0.693/g is marked in the Figure at 1, 2, and 3 years such that the proportion of patients with slower doubling times than those thresholds can be readily seen.

**Fig. 6:**
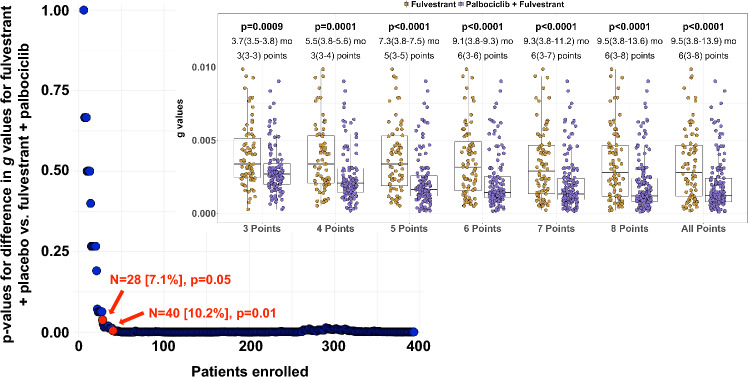
Simulation demonstrating the small number of patients needed to achieve a statistically valid difference between the ***g*** values of the two treatment arms. The figure in the upper right-hand corner shows that with as few as three measurements (baseline plus two on-study imaging studies), statistical differences in ***g*** values could be discerned. The number of months refers to the amount of time (in months) elapsed between the baseline scan and *N*th scan. In each column, all patients with available data who had undergone a baseline scan plus at least two additional scans were included. For example, the first column, labeled “3 points”, includes the data of all patients who have undergone a baseline scan plus two additional scans. The second column, labeled “4 points”, includes the data of all patients who have undergone a baseline scan plus up to three additional scans, *including* those who were included in the previous column (i.e., baseline plus two additional scans), hence the label (3-*N*) points. Turning to the order in which patients were enrolled, one can see that differences in g that were statistically significant could be achieved with as few as 28 patients [19 randomized to palbociclib and 9 to placebo] and 40 [27 randomized to palbociclib and 13 to placebo] with *p*-values of 0.05 and 0.012, respectively

A benefit of our regression-growth model is that it treats tumor burden as a continuous variable, a valuable attribute given that prior studies have demonstrated significant reduction in the sample size needed to power a two-arm study when a continuous rather than a dichotomous endpoint is used [34, 35]. Such a possibility is confirmed by the data shown in Fig. 6. The graph in the upper right looks at differences between the two arms of the trial with incremental increases in the number of data points available. Statistically significant differences in ***g*** values could be discerned with as few as three data points, i.e., the baseline and two follow-up values six weeks apart. Given this, we asked how many patients one would need to enroll before ***g*** values of the two arms could be confirmed to be statistically different. As few as 28 [19 randomized to palbociclib and 9 to placebo] and 40 [27 randomized to palbociclib and 13 to placebo] patients achieved p-values of 0.05 and 0.012, respectively. In practical terms this would have happened in May 2014 by which time the first 10.4% of patients who enrolled had undergone their second post-enrollment reassessment. This would have been 9 months before palbociclib was granted accelerated approval, 2 years before it received full approval, and 7 years before—at a median 73 months of follow-up [20, 21]—the study achieved a statistical difference in OS, an outcome consistent with the differences in ***g*** values. Having established ***g*** as a biomarker of OS across a range of malignancies, we would envision a scenario where an early demonstration of a statistically meaningful difference in ***g*** between the control and experimental arms could either support an accelerated approval to be converted to a full approval when more conventional endpoints are secured, or alternately provide a completed confirmatory trial for full approval. Ultimately, the use of ***g*** as an endpoint to inform OS requires prospective validation. We would note this was the conclusion reached by the USFDA following the analysis of data from nearly 10,000 patients with NSCLC [36]. A validation study could be designed as a parallel analysis—with ***g*** as the endpoint being tested, and PFS and OS the conventional endpoint. Recommendations for study continuation or discontinuation based on statistically significant differences in ***g*** between arms would be made at predetermined intervals, but not acted on in a validation study.

The method of analysis we describe here also has the potential to provide information that could help guide patient-level clinical decision-making. Specifically, in a patient with metastatic HR-positive, HER2-negative breast cancer in whom a curative option is not available, the approach we describe could provide the information needed to ensure that a therapy that is well-tolerated and may be bringing benefit by slowing tumor growth is not abandoned. For example, in renal cell carcinoma we reported prolonged stability of the growth rate, g, in patients receiving sunitinib, suggesting that despite an increase in tumor size, sunitinib was still controlling the growth rate [37].

A similar concept could be envisioned for the patient population studied here. Patients with slowly increasing tumor size could be maintained on palbociclib if the growth rate were found to be low and slow. As to what value of ***g*** might be acceptable for continuing palbociclib, we would suggest that, just as what duration of stable disease is generally deemed valuable [38], so too could a consensus be reached as to a ***g*** value acceptable for continuing a therapy. ***g*** values can be readily converted to doubling times (DT) by dividing the natural log of 2 (0.693) by ***g***. On the palbociclib arm, ***g*** values of 0.0019, 0.00095 and 0.00063 or below, representing DTs of 1, 2 and 3 years, respectively, were achieved in 52%, 29% and 11% and in 45%, 25% and 14% of women with fulvestrant-sensitive and fulvestrant-resistant disease, respectively (Supplemental Fig. 8, Supplemental Table 2). Any value smaller than the chosen threshold ***g*** (for example, ***g*** = 0.00095, equivalent to a doubling time of more than 2 years) would then be considered an acceptable rate of tumor growth to continue the current line of treatment.

We acknowledge that our study has limitations. These include insufficient data leading to exclusion of a small subset of patients, exclusion of tumor data that cannot be fit by any of the four equations, and inter-reader variability for radiographic tumor measurement data, although we were gratified that in this analysis, those were few.

In summary we have successfully estimated the rates of tumor growth, ***g***, in PALOMA-3. Our results now add breast cancer to the list of tumors in which we have been able to demonstrate ***g*** is a biomarker of OS. Our analysis confirms the reported outcomes in PALOMA-3 demonstrating a meaningful prolongation of OS with palbociclib with a median OS advantage of 6.9 months in this trial. We further confirm that amongst this more difficult to treat patient population, the addition of palbociclib was effective in patients with endocrine-sensitive disease. Because*** g*** is estimated while a patient is enrolled in a clinical trial, long before traditional endpoints mature and can be analyzed, these results argue strongly for using ***g*** as a clinical trial endpoint to help inform go/no-go decisions, improve trial efficiency, and deliver novel therapies to patients sooner.

### Supplementary Information

Below is the link to the electronic supplementary material.

Supplementary file1 (PPTX 4847 KB)

## Data Availability

All data will be shared for academic pursuits.
