# Supplementary material for: Analysis of data from the PALOMA-3 trial confirms the efficacy of palbociclib and offers alternatives for novel assessment of clinical trials
Source: Breast Cancer Res Treat. 2023 Nov 13;204(1):39–47. doi: 10.1007/s10549-023-07131-7 (PMC10805865; doi:10.1007/s10549-023-07131-7)
Supplement: Supplementary file 1 — Supplementary file1 (PPTX 4847 KB) [file 10549_2023_7131_MOESM1_ESM.pptx]

## Slide 1
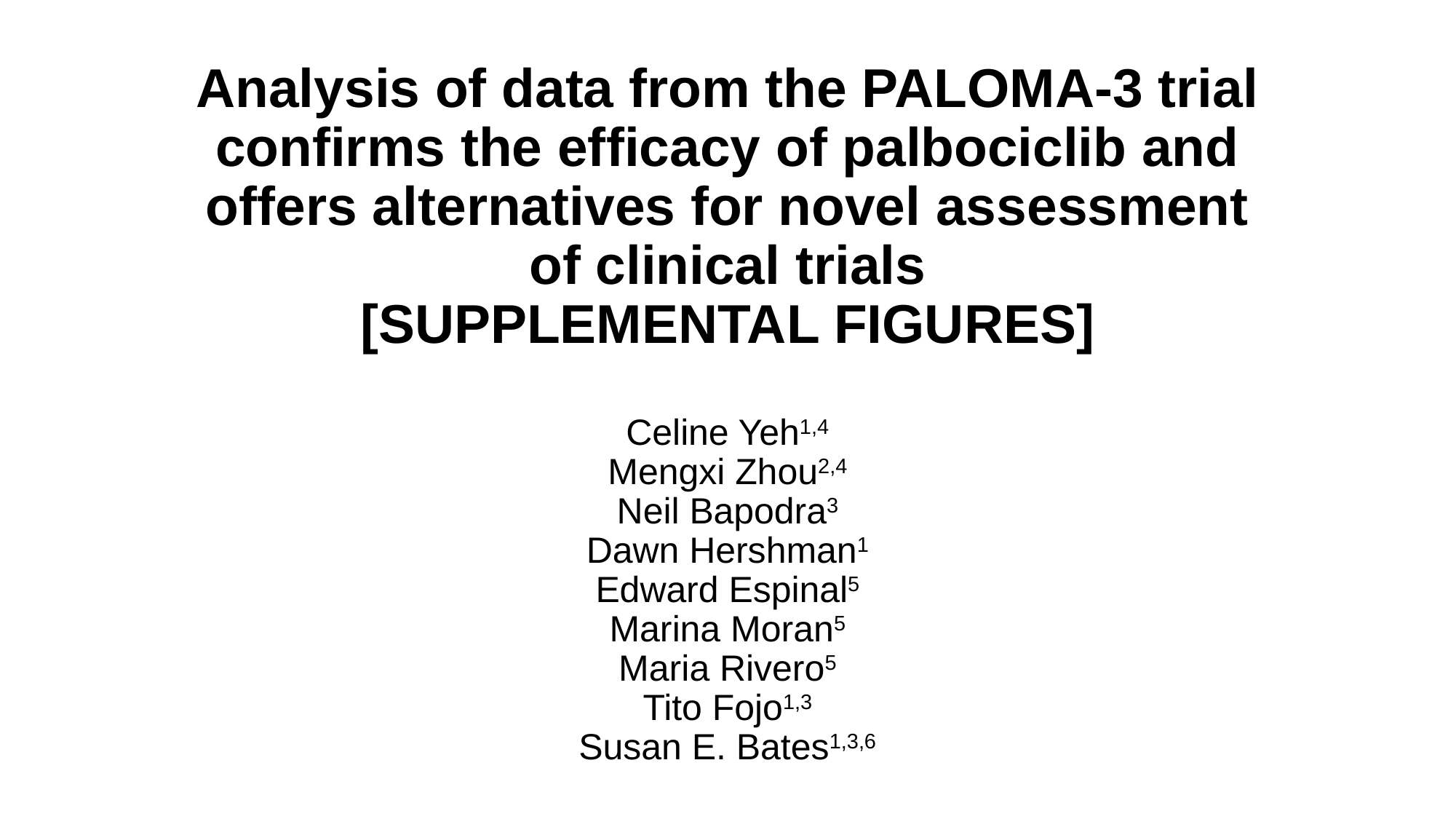

# Analysis of data from the PALOMA-3 trial confirms the efficacy of palbociclib and offers alternatives for novel assessment of clinical trials [SUPPLEMENTAL FIGURES]   Celine Yeh1,4 Mengxi Zhou2,4 Neil Bapodra3 Dawn Hershman1 Edward Espinal5 Marina Moran5 Maria Rivero5 Tito Fojo1,3 Susan E. Bates1,3,6

## Slide 2
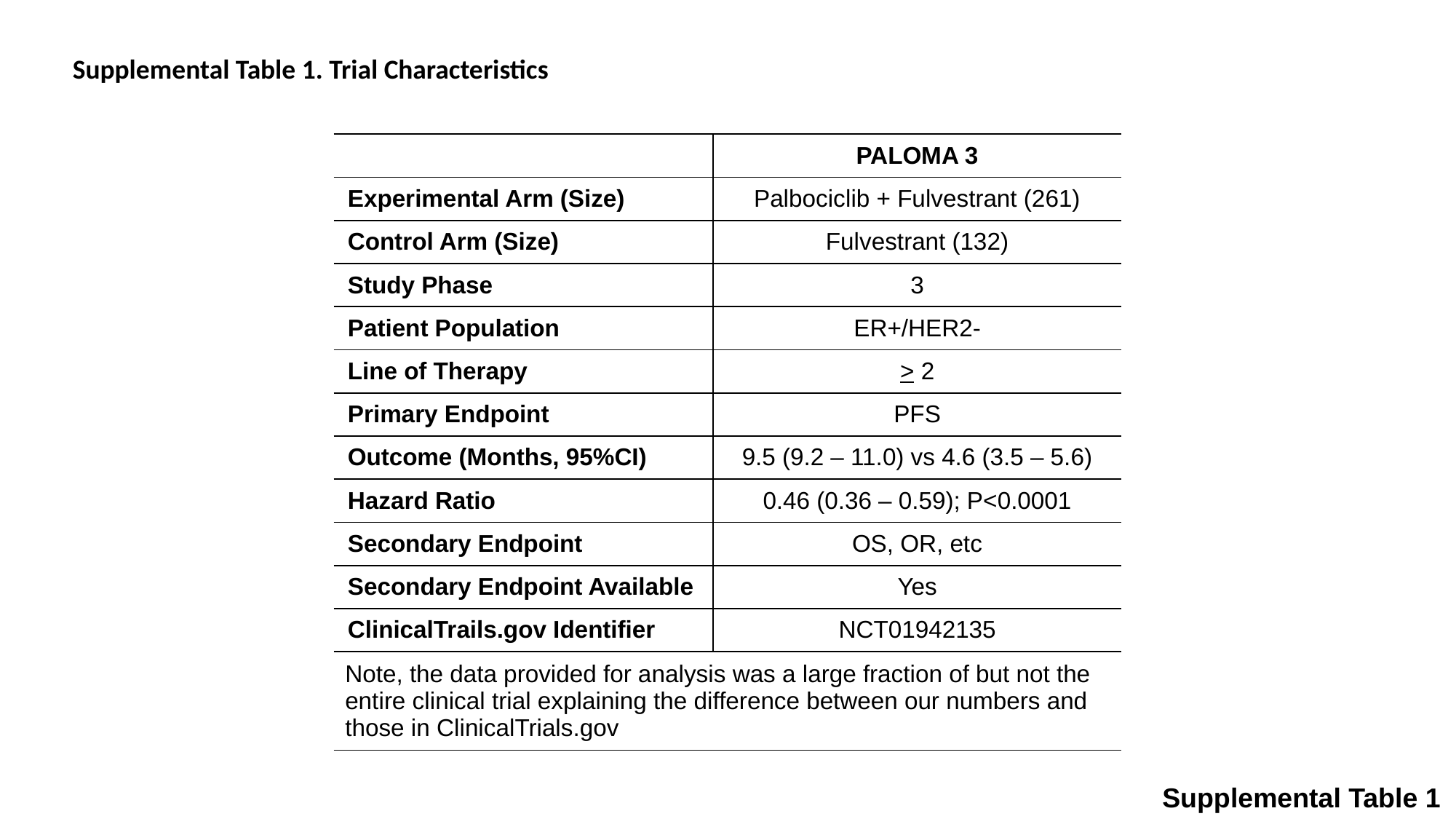

Supplemental Table 1. Trial Characteristics
| | PALOMA 3 |
| --- | --- |
| Experimental Arm (Size) | Palbociclib + Fulvestrant (261) |
| Control Arm (Size) | Fulvestrant (132) |
| Study Phase | 3 |
| Patient Population | ER+/HER2- |
| Line of Therapy | > 2 |
| Primary Endpoint | PFS |
| Outcome (Months, 95%CI) | 9.5 (9.2 – 11.0) vs 4.6 (3.5 – 5.6) |
| Hazard Ratio | 0.46 (0.36 – 0.59); P<0.0001 |
| Secondary Endpoint | OS, OR, etc |
| Secondary Endpoint Available | Yes |
| ClinicalTrails.gov Identifier | NCT01942135 |
| Note, the data provided for analysis was a large fraction of but not the entire clinical trial explaining the difference between our numbers and those in ClinicalTrials.gov | |
Supplemental Table 1

## Slide 3
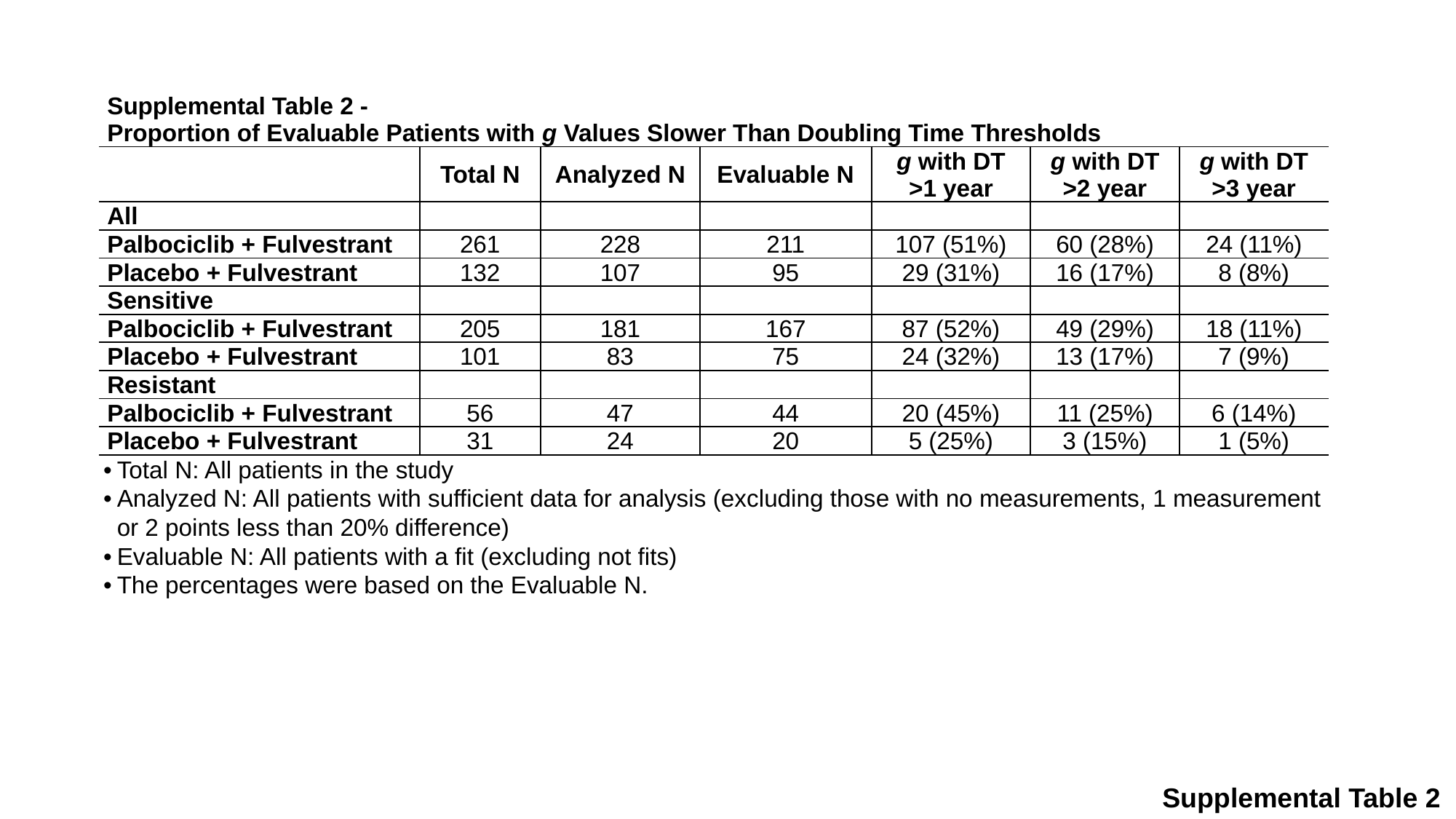

| Supplemental Table 2 - Proportion of Evaluable Patients with g Values Slower Than Doubling Time Thresholds | | | | | | |
| --- | --- | --- | --- | --- | --- | --- |
| | Total N | Analyzed N | Evaluable N | g with DT >1 year | g with DT >2 year | g with DT >3 year |
| All | | | | | | |
| Palbociclib + Fulvestrant | 261 | 228 | 211 | 107 (51%) | 60 (28%) | 24 (11%) |
| Placebo + Fulvestrant | 132 | 107 | 95 | 29 (31%) | 16 (17%) | 8 (8%) |
| Sensitive | | | | | | |
| Palbociclib + Fulvestrant | 205 | 181 | 167 | 87 (52%) | 49 (29%) | 18 (11%) |
| Placebo + Fulvestrant | 101 | 83 | 75 | 24 (32%) | 13 (17%) | 7 (9%) |
| Resistant | | | | | | |
| Palbociclib + Fulvestrant | 56 | 47 | 44 | 20 (45%) | 11 (25%) | 6 (14%) |
| Placebo + Fulvestrant | 31 | 24 | 20 | 5 (25%) | 3 (15%) | 1 (5%) |
| Total N: All patients in the study Analyzed N: All patients with sufficient data for analysis (excluding those with no measurements, 1 measurement or 2 points less than 20% difference) Evaluable N: All patients with a fit (excluding not fits) The percentages were based on the Evaluable N. | | | | | | |
Supplemental Table 2

## Slide 4
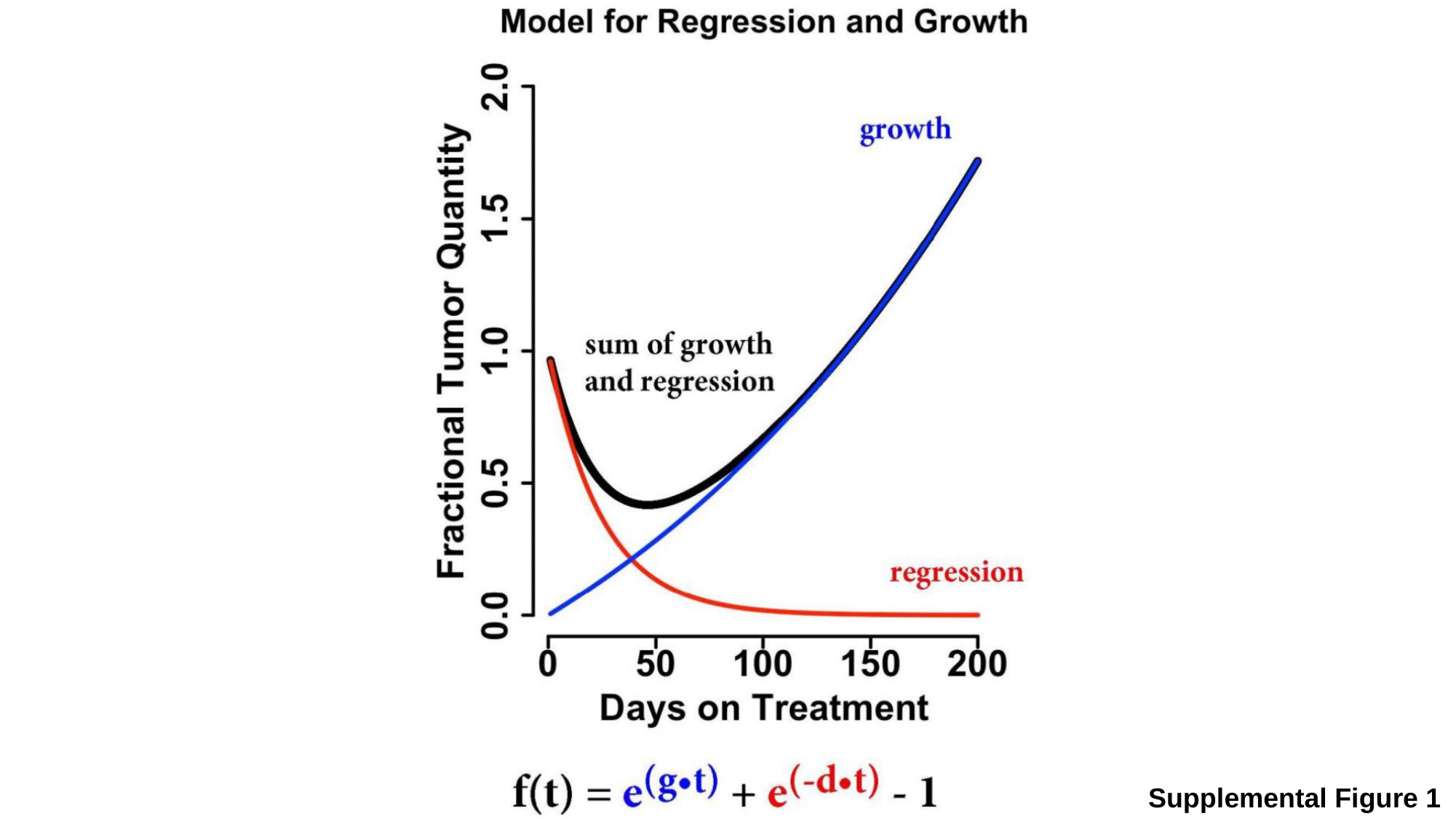

Supplemental Figure 1

## Slide 5
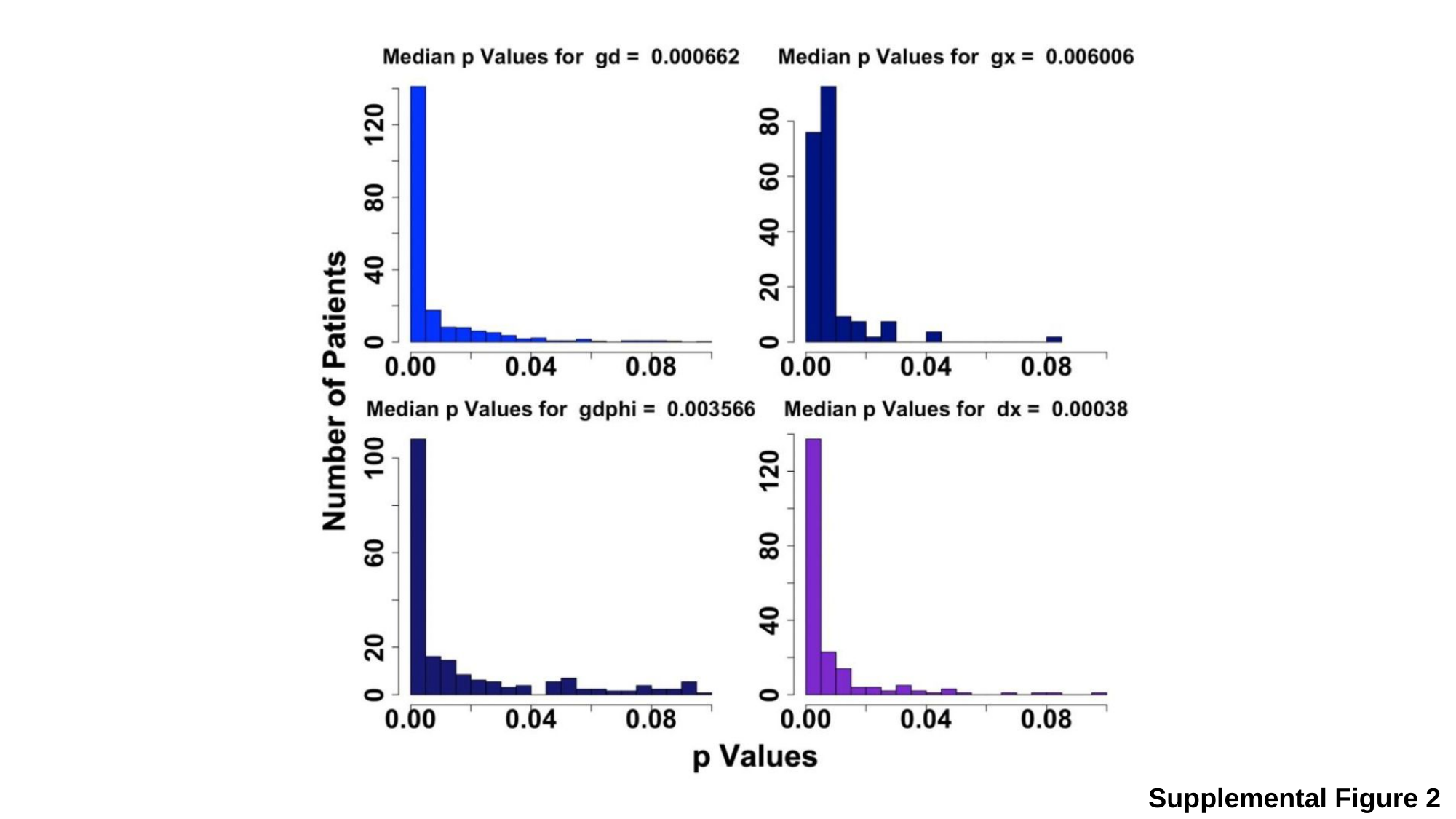

Supplemental Figure 2

## Slide 6
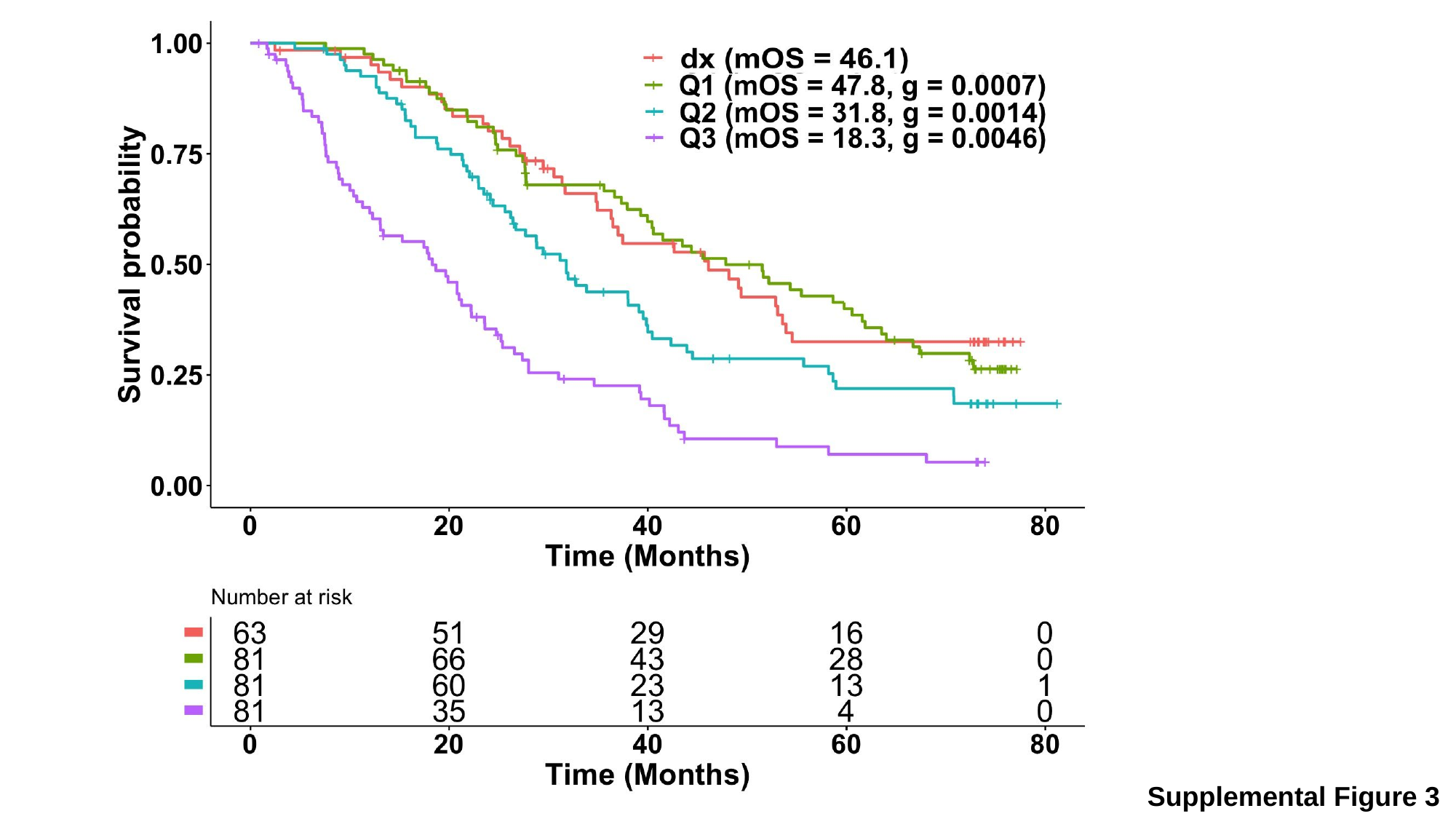

Supplemental Figure 3

## Slide 7
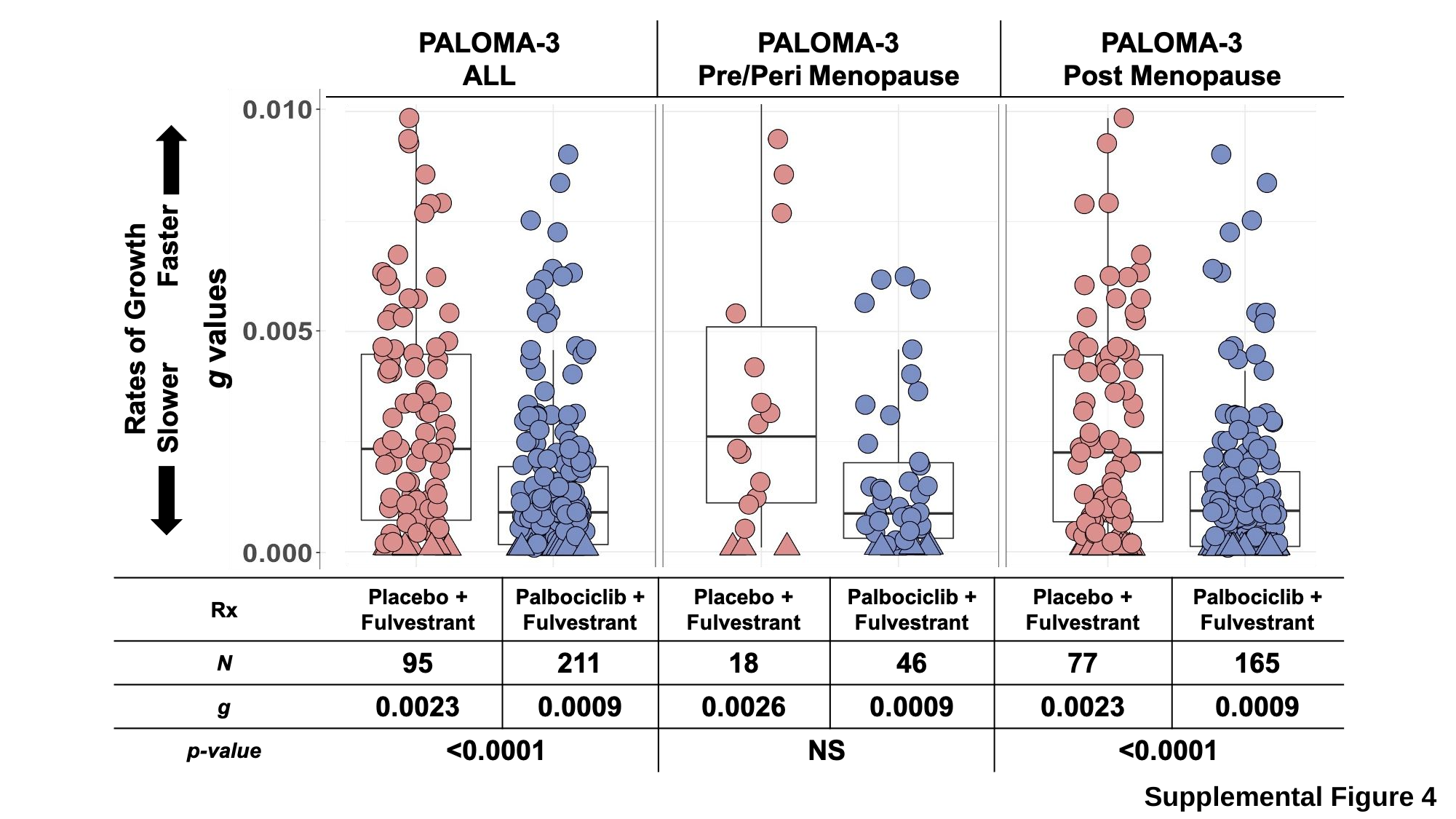

Supplemental Figure 4

## Slide 8
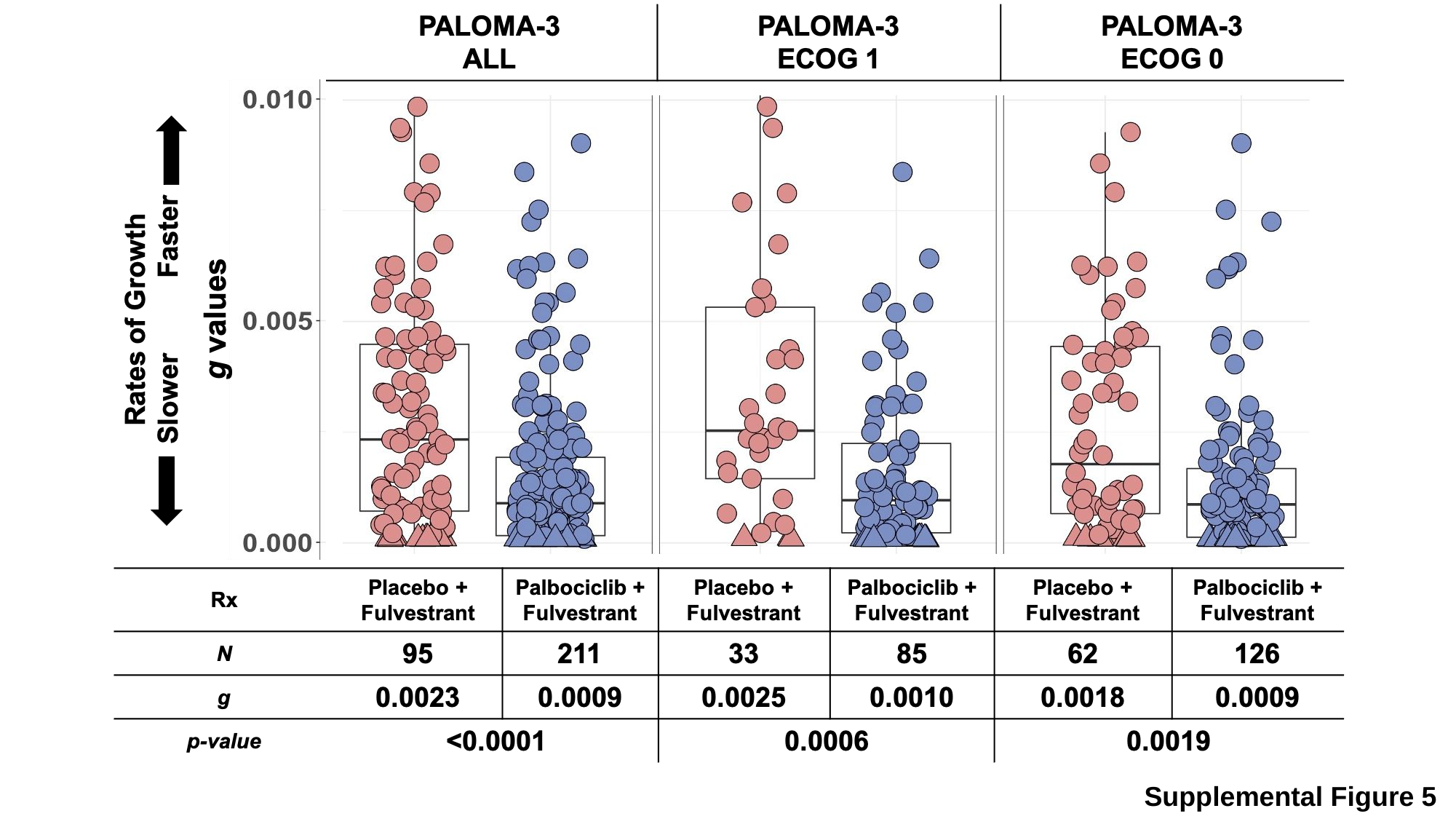

Supplemental Figure 5

## Slide 9
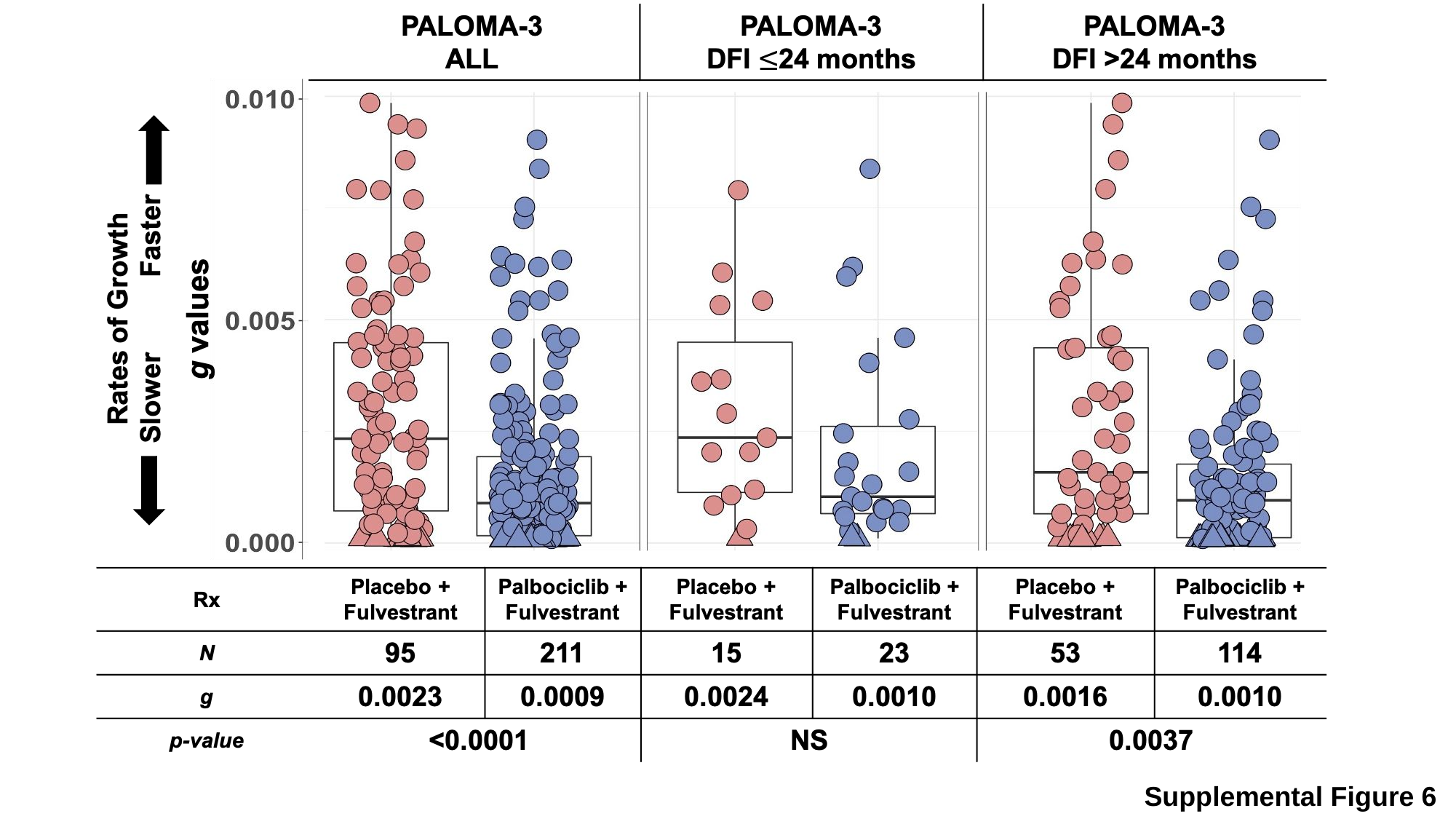

Supplemental Figure 6

## Slide 10
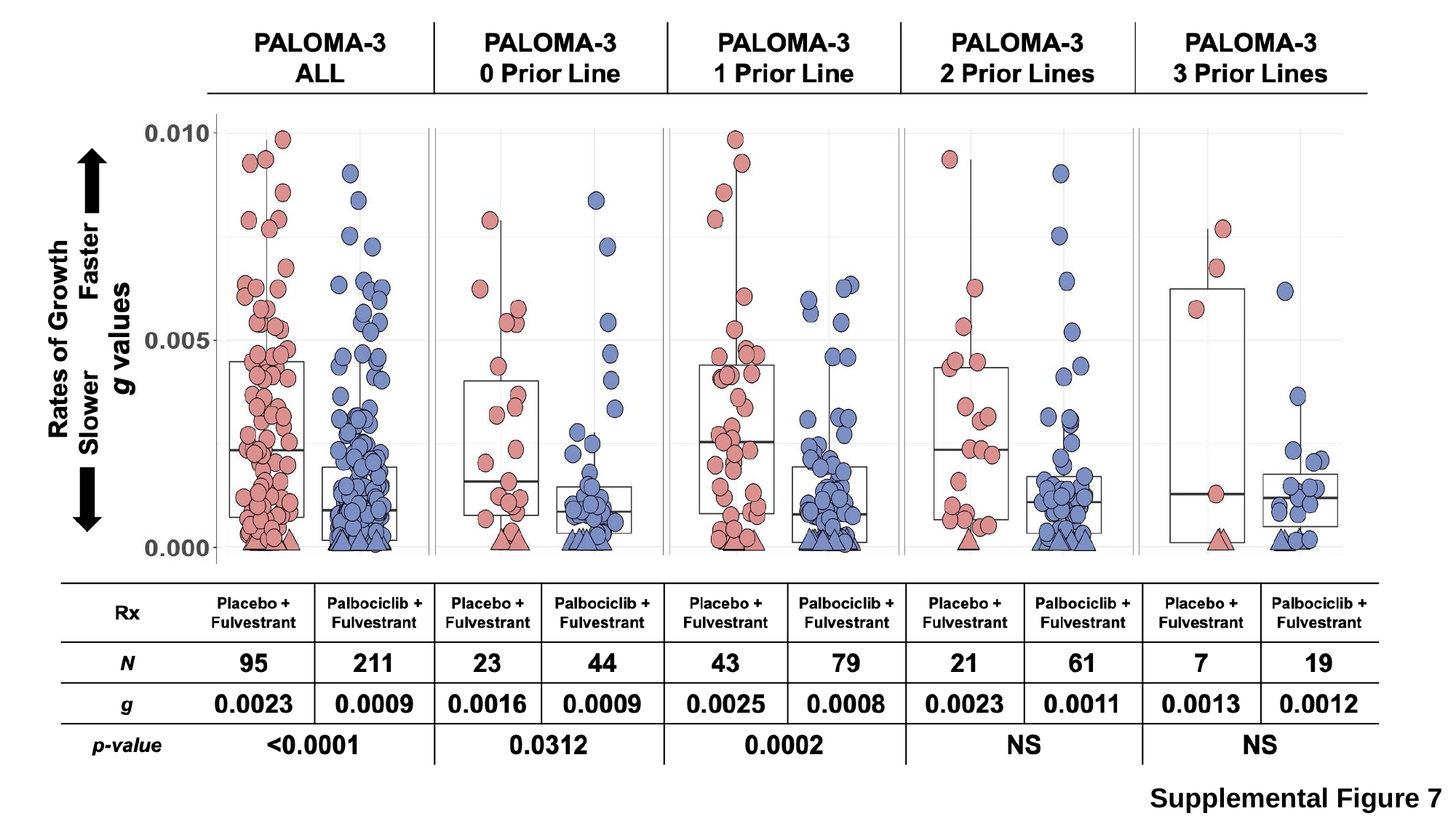

Supplemental Figure 7

## Slide 11
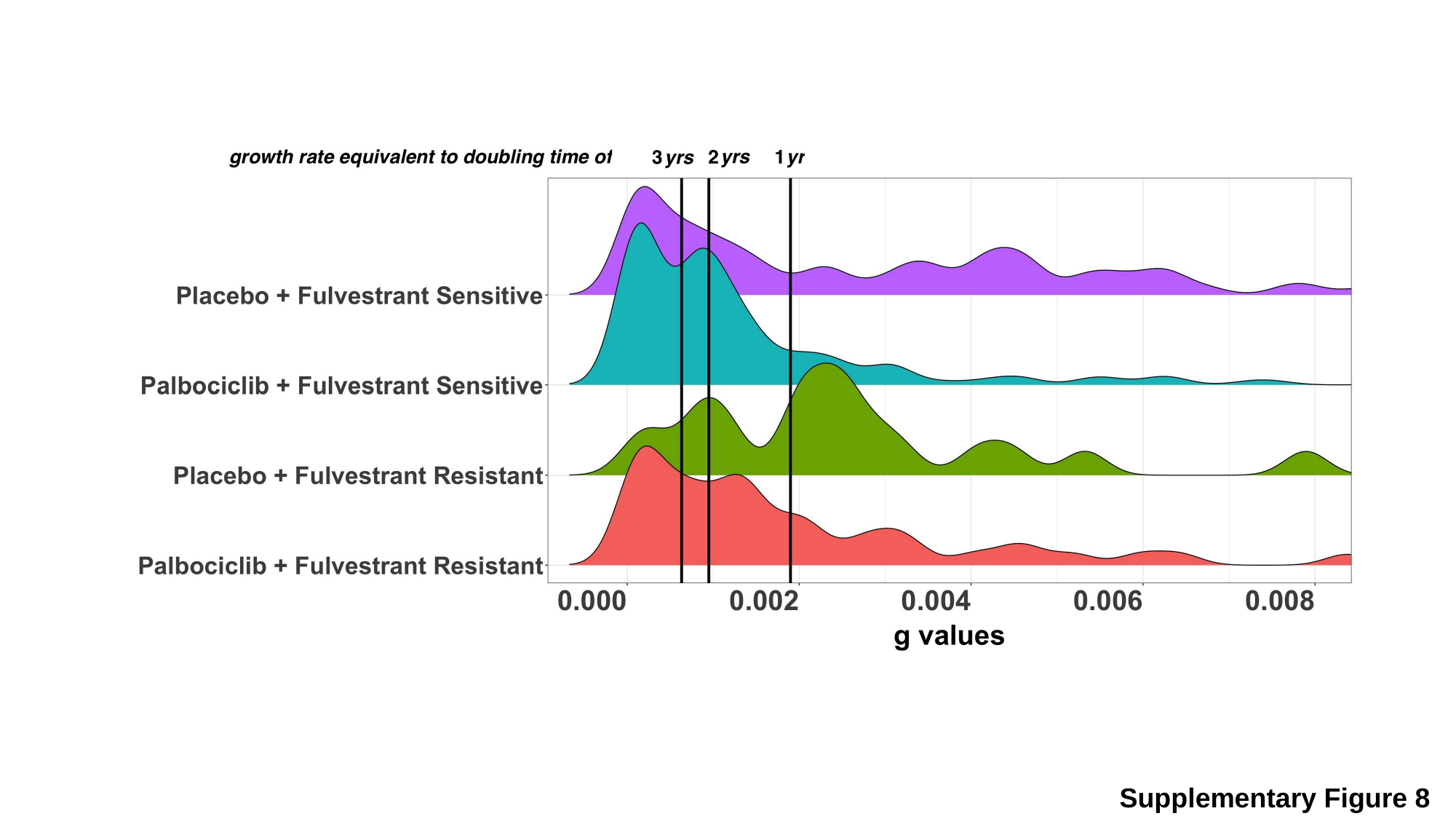

Supplementary Figure 8
